# Case Report: The Forgotten Functions of the Hindbrain

**DOI:** 10.1192/bjo.2025.10759

**Published:** 2025-06-20

**Authors:** Shalina Ramsewak, Shruti Lodhi

**Affiliations:** Surrey and Borders Partnership NHS Trust, Camberley, United Kingdom

## Abstract

**Aims:** This case highlights the cognitive functions of the cerebellum which is often forgotten about.

**Methods:** An 80-year-old lady with a previous mental health diagnosis of Bipolar Affective disorder (BIPAD). She presented to the general hospital in July 2024 with a sudden onset of confusion and aggressive behaviour. She was referred to the Psychiatric Liaison Service (PLS). On assessments, she presented with no clear mood or psychotic disorder suggestive of relapse in her BIPAD. She scored 8 out 30 on the Mini-Addenbrooke’s Cognitive Examination (M-ACE) – losing marks on attention, memory – registration/recall, verbal fluency and the clock drawing test showed neglect. She lacked insight into her presentation and thus was in hospital under Deprivation of Liberty Safeguards (DoLS). Collateral history from her son corroborated that this was not a relapse in BIPAD. He enquired about an MRI head which revealed a small old infarct in the left cerebellar hemisphere.

**Results:** Cerebellar cognitive affective syndrome (CCAS; Schmahmann’s syndrome) would explain the timeline of symptoms, assessment findings and collateral history. CCAS is characterized by deficits in executive function, linguistic processing, spatial cognition, and affect regulation. Neuropsychiatric features include impairments in attentional control, emotional control, psychosis spectrum disorders and social skill set. The deficits suggest a disruption of the cerebellar modulation of neural circuits that link frontal, parietal, temporal, and limbic cortices with the cerebellum which is known as Cerebellum-Cerebrum cortex loop. Movement, co-ordination, and cognition are intricately connected within the brain, however the role of the cerebellum in cognition has often been ignored.

**Conclusion:** Clinicians tend to focus on the motor-coordination functions of the hindbrain, and this case report highlights cognitive functions of the hindbrain that are often forgotten. CCAS may be underdiagnosed due to the lack of awareness of the intricate connections between the hindbrain and forebrain through the cerebellum-cerebrum cortex loop, responsible for cognitive function within the hindbrain. This can lead to inappropriate treatment plans being devised for patients, and subsequent negative impact on management outcomes and even quality of life.

